# Association of Weight Loss in Ambulatory Care Settings With First Diagnosis of Lung Cancer in the US

**DOI:** 10.1001/jamanetworkopen.2023.12042

**Published:** 2023-05-11

**Authors:** Larry G. Kessler, Brian D. Nicholson, Hannah A. Burkhardt, Jason Oke, Matthew J. Thompson

**Affiliations:** 1 Department of Health Systems and Population Health, School of Public Health, University of Washington, Seattle; 2 Oxford University, Oxford, United Kingdom; 3 Department of Biomedical Informatics and Medical Education, University of Washington, Seattle; 4 Department of Family Medicine, University of Washington, Seattle

## Abstract

**Importance:**

Lung cancer, the US’s leading cause of cancer death, is often diagnosed following presentation to health care settings with symptoms, and many patients present with late-stage disease.

**Objective:**

To investigate the association between weight loss and subsequent diagnosis of incident lung cancer in an ambulatory care population and to assess whether recorded weight change had higher odds of lung cancer diagnosis than objective measurements.

**Design, Setting, and Participants:**

This case-control study included patients visiting a US academic medical center between January 1, 2012, and December 31, 2019. Data were derived from US ambulatory care electronic health records from the University of Washington Medical Center linked to the local Surveillance, Epidemiology, and End Results cancer registry. Cases were identified from patients who had a primary lung cancer diagnosis between 2012 and 2019; controls were matched on age, sex, smoking status, and presenting to the same type of ambulatory clinic as cases. Data were analyzed from March 2022 through January 2023.

**Exposure:**

Continuous and categorical weight change were assessed.

**Main Outcomes and Measures:**

Odds ratios estimating the likelihood of a diagnosis of lung cancer were calculated using univariable and multivariable conditional logistic regression.

**Results:**

A total of 625 patients aged 40 years or older with a first primary lung cancer diagnosis and 4606 matched controls were included (1915 [36.6%] ages 60 to 69 years; 418 [8.0%] Asian, 389 [7.4%] Black, 4092 [78.2%] White). In unadjusted analyses, participants with weight loss of 1% to 3% (odds ratio [OR], 1.12; 95% CI, 0.88-1.41), 3% to 5% (OR, 1.36; 95% CI, 0.99-1.88), or 5% to 10% (OR, 1.23; 95% CI, 0.82-1.85) over a 2-year period did not have statistically significantly increased risk of lung cancer diagnosis compared with those who maintained a steady weight. However, participants with weight loss of 10% to 50% had more than twice the odds of a lung cancer diagnosis (OR, 2.27; 95% CI, 1.27-4.05). Most categories of weight loss showed significant associations with an increased risk of lung cancer diagnosis for at least 6 months prior to diagnosis. Patients who had weight loss both recorded in clinicians’ notes and measured had higher odds of lung cancer compared with patients who had only recorded (OR, 1.26; odds; 95% CI, 1.04-1.52) or measured (OR, 8.53; 95% CI, 6.99-10.40) weight loss.

**Conclusions and Relevance:**

In this case-control study, weight loss in the prior 6 months was associated with incident lung cancer diagnosis and was present whether weight loss was recorded as a symptom by the clinician or based on changes in routinely measured weight, demonstrating a potential opportunity for early diagnosis. The association between measured and recorded weight loss by clinicians presents novel results for the US.

## Key Points

**Question:**

Is weight loss associated with a diagnosis of lung cancer, whether this loss is recorded by primary care clinicians or by objective measurements?

**Findings:**

This case-control study analyzed data from 2012 through 2019 on 625 patients aged 40 years or older with a first primary lung cancer diagnosis, and 4606 controls matched by age, sex, and smoking status in a US ambulatory care medical system. Patients with lung cancer diagnosis had statistically significant greater odds of weight loss in the previous 6 months than matched controls.

**Meaning:**

These results suggest that weight loss may be associated with incident lung cancer among patients, demonstrating a potential opportunity for early diagnosis.

## Teaser

This case-control study of US adults examines the association of weight loss with future lung cancer diagnosis.

## Introduction

Lung cancer is the third most common cancer and the leading cause of cancer death in the US.^1^ Even though lung cancer screening has been shown effective in multiple randomized trials of low-dose computed tomography,^2,3^ screening uptake remains low in the US.^4^ Most patients are diagnosed with lung cancer following presentation to health care settings with symptoms, and many patients (47%) present with late-stage disease (stages 3 or 4).^5^

Diagnosing lung cancer following presentation with nonspecific symptoms such as weight loss, loss of appetite, or fatigue is challenging, as these symptoms are more commonly associated with benign conditions or may be overlooked for long periods of time.^6^ This contrasts with specific symptoms such as hemoptysis, cough, and shortness of breath, for which the site of origin to investigate is clearer. Unlike other symptoms associated with lung cancer, weight loss can both be noted subjectively by patients as a symptom, and also identified objectively as a clinical sign, especially as weight is routinely measured at visits to ambulatory care health facilities in the US.^7^ Moreover, the amount and trajectory of measured weight change over time may provide evidence of a signal to alert primary care clinicians to the possibility of cancer (or other conditions associated with weight loss). Previous studies have noted significant associations between weight loss and several cancers, but evidence for an association for lung cancer is not clear.^7,8^ Improving the speed and accuracy of detecting lung cancer in primary care has the opportunity to potentially reduce morbidity from the challenging and invasive treatments for lung cancer, and possibly improve outcomes.^9,10^ In this study we aimed to identify evidence for associations between weight loss, identified from coded data, free-text data, and routine clinic measurements, and lung cancer in an ambulatory care population.

## Methods

### Study Design

We performed a case-control study using data from the University of Washington Medicine (UWM) electronic health records (EHR) and the Seattle/Puget Sound Surveillance, Epidemiology, and End Results (SEER) Program, a National Cancer Institute–supported national cancer registry. This study was approved by the University of Washington Human Subjects Division. Informed consent was not required as these data were deidentified and this was a secondary analysis of medical record data. The study was reported in line with the Strengthening the Reporting of Observational Studies in Epidemiology (STROBE) reporting guideline.

### Setting

Cases and controls received ambulatory care at UWM, a large academic health center that provides primary through tertiary care. We identified these study participants using the UWM enterprise-wide data warehouse.

### Study Population

Cases were identified from UWM patients aged 40 years or older, with a first primary lung cancer diagnosis between January 1, 2012, and December 31, 2019, who had an established relationship with a UWM ambulatory care setting in the 2 years before the date of their first recorded lung cancer *International Classification of Diseases, Ninth Revision *(*ICD-9*) or *International Statistical Classification of Diseases and Related Health Problems, Tenth Revision *(*ICD-10*) code (EHR diagnosis date).^11^ For each case, 10 controls were individually matched on age, sex (male, female), and smoking status (ever vs never), if they presented to the same type of ambulatory care clinic within 3 months of the lung cancer diagnosis date. For the current study, we excluded 14 cases and 7 controls for whom we had any record at UWM of gastric band surgery. Other than the bariatric surgery exclusion, we did not select controls based on any other diagnostic criteria as we wanted as broad a spectrum as possible of patients visiting these same clinics as the cases. Given that our aim is to determine if symptoms might be recognized even in the presence of a likely non–lung cancer diagnosis among general population visiting ambulatory care settings, this approach minimizes any diagnosis-related bias.

We report both race and ethnicity and their association with weight loss to define our population for readers. Categories for race included American Indian or Alaska Native, Asian, Black or African American, Native Hawaiian or other Pacific Islander, White, multiple races, and unknown. These variables were derived from the EHR and were self-reported at UWM.

### Data Preparation

#### Extraction

An EHR data extraction protocol was applied to all encounters in the 2-year period prior and up to 6 months following the diagnosis date (cases) and index date (controls). These data comprised demographics, all *ICD-10* codes and *Common Procedural Terminology* procedure codes linked to encounters, and quantitative weight observations (measurements).

We identified recorded weight loss in the coded data (using *ICD-9* code 783.21 and *ICD-10* code R63.4) and unstructured (free-text) notes data using a previously reported event-based natural language processing (NLP) pipeline.^12,13^ To facilitate combination with coded data, which provides information on present but not absent symptoms, negated symptoms extracted by the NLP tool were not included. Weight measurements were extracted and converted to kilograms (kg) from any health care encounters where these were recorded in the 2 years prior to the diagnosis or index date.

### Filtering

We removed cases and matched controls when implausible weight measurements were identified. When more than 1 measurement was recorded on a single day they were replaced with their average.

#### Observed Weight Loss Episodes

Measured weight change episodes were defined as the period between any 2 adjacent weight measurements, measured in days, and presented as both percentage change (continuous variable) and as the following categorical variable: over 1% to 50% (ie, weight gain of over 1%), less than 1% weight to 1% weight gain (ie, stable weight), less than 3% to 1% weight loss, less than 5% to 3% weight loss, less than 10% to 5% weight loss, and less than 10% to 50% weight loss (ie, weight loss of 10% or more).

#### Normalized Weight Loss Episodes

Previous studies have demonstrated that unexpected weight loss occurring at periods not proximate to the diagnosis of cancer are also associated with this eventual diagnosis.^14^ In order to compare weight changes between diagnosis and specific prior time points of interest, we used straight-line interpolation of each person’s weight at day of diagnosis, as well as each of 8 quarters before diagnosis. In this manner, we determined each patient’s weight at 90-day intervals (up to 720 days) before diagnosis. We defined 8 weight loss episode categories with a length of 90 days each. Patients were lost for an individual quarter if they did not have measurements both inside and outside each quarter boundary.

### Statistical Analysis

In the primary analysis, we estimated the association between weight loss and a subsequent diagnosis of lung cancer using a range of weight loss definitions. We used nominal 95% CIs for the odds ratios (ORs) to determine significance. The secondary analysis compared the estimated value of using weight measurements to define weight loss with weight loss documented either as an *ICD-9* or *ICD-10* code or in free text of the EHR. Analysis was conducted with Python version 3.7 and Statsmodels version 0.13.2.

#### Odds of a Lung Cancer Diagnosis

Univariate and multivariate ORs were calculated using conditional logistic regression (conditioned on matching group). To address within-patient correlation, we reduced the data set to a single measurement per person by randomly selecting 1 weight loss episode per patient. Analyses were repeated for both percentage weight change (continuous) and weight change categories, in which the reference was the group of participants who maintained stable weight (ie, 1% weight change in either direction). Regression models were run first without further adjustments (beyond the study design and matching). We then adjusted for the interval over which weight change occurred (ie, the number of days between weight observations) and for the baseline weight (first recorded weight within the 24-month period).

#### Weight Changes Across Specific Time Intervals of Interest

To identify opportunities for early detection and to account for acute effects immediately before diagnosis, we repeated analyses with all weight loss data from within 1 month and within 3 months of diagnosis removed. For these analyses, we repeated the process of randomly sampling 1 episode using all data from the restricted time period.

We repeated the analysis with the normalized weight loss episodes, calculated from interpolated patient weights, for each time interval of interest (weight change within the first, second, and up to the eighth quarter before diagnosis). A separate regression was run for each quarter, adjusted for patient baseline weight.

#### Estimated Values of Measured vs *ICD* or NLP Weight Loss

Our secondary analysis investigated the correlation between measured weight loss and clinicians’ documentation of weight loss as a code or free text, and the estimated value for lung cancer associated with each method of documenting weight loss. We dichotomized measured weight change data to align with the binary nature of the coded and free-text data. We derived projections from ORs for each definition of weight loss, adjusting for weight loss being present per the other data source, and entered both of these variables in the same multivariable regression model. The analysis was repeated excluding data from the 1 month immediately before diagnosis, as well as the 3 months immediately before diagnosis.

## Results

### Observations for Analysis

Of 689 cases and 6746 controls that met inclusion criteria, 64 cases and 2140 controls were excluded due to insufficient weight data. Our data included 625 confirmed cases of lung cancer with at least 2 weight measurements and 4606 matched controls (Table 1). Approximately 20% of the participants were under age 60 years (991 of 5231 participants), the largest subcohort by decade was aged between 60 and 69 years (1915 participants [36.6%]); 2512 participants (48.0%) were female, 418 [8.0%] Asian, 389 [7.4%] Black, and 4092 [78.2%] White. Age and sex information was available for all individuals; race and ethnicity were missing for 213 (4.0%) and 428 (8.1%) individuals, respectively. There were more weight measurements in cases (mean [SD], 13.1 [11.9]) than controls (9.7 [11.7]) (Table 1), reflecting the higher average number of clinical visits in the 3 months before a lung cancer diagnosis (mean [SD] visits: cases, 22.9 [15.5] vs controls, 7.4 [13.0]). Out of 8 possible quarterly weight loss episodes (across our 24-month period), cases and controls had data for a mean (SD) of 5.7 (2.0) and 5.2 (2.1) quarters, respectively.

**Table 1.** Demographics of Participants

Characteristics	Participants, No. (%)	
	Cases (n = 625)	Controls (n = 4606)
Age, y		
<60	134 (21.4)	857 (18.6)
60-69	232 (37.1)	1683 (36.5)
70-79	170 (27.2)	1346 (29.2)
≥80	89 (14.2)	720 (15.6)
Race		
American Indian or Alaska Native	6 (1.0)	44 (1.0)
Asian	65 (10.4)	353 (7.7)
Black or African American	62 (9.9)	327 (7.1)
Native Hawaiian or other Pacific Islander	3 (0.5)	29 (0.6)
White	473 (75.7)	3619 (78.6)
Multiple races	5 (0.8)	32 (0.7)
Unknown	11 (1.8)	202 (4.4)
Ethnicity		
Hispanic or Latino	20 (3.2)	156 (3.4)
Not Hispanic or Latino	567 (90.7)	4060 (88.1)
Unknown	38 (6.1)	390 (8.5)
Sex		
Female	303 (48.5)	2209 (48.0)
Male	322 (51.5)	2397 (52.0)
Weight measurements, No.		
Mean (SD)	13.1 (11.9)	9.7 (11.7)
Median (IQR)	9.0 (5.0-17.0)	6.0 (3.0-11.0)
Range	2.0-102.0	2.0-211.0

### Odds of a Lung Cancer Diagnosis

Univariate and multivariate results in Table 2 show a distinct gradient of point estimates from weight gain to higher levels of weight loss. In unadjusted analyses, individuals with weight loss of 1% to 3% (odds ratio [OR], 1.12; 95% CI, 0.88-1.41), 3% to 5% (OR, 1.36; 95% CI, 0.99-1.88), or 5% to 10% (OR, 1.23; 95% CI, 0.82-1.85) over a 2-year period did not have statistically significantly increased risk of lung cancer diagnosis compared with individuals who maintained a steady weight. However, patients with weight loss of 10% to 50% had more than twice the odds of a lung cancer diagnosis (OR, 2.27; 95% CI, 1.27-4.05). For smokers, the odds of being a case continued to increase substantially even with the time intervals most proximate to the diagnosis were removed.

**Table 2.** Odds of Lung Cancer Diagnosis Among Patients by Continuous and Categorical Weight Change

Characteristic	OR (95% CI)		
	Entire 24 mos prior to diagnosis (or index date)	Excluding 1 mo prior to diagnosis (or index date)	Excluding 3 mos prior to diagnosis (or index date)
Unadjusted odds by weight change category			
>−1% to ≤1%	1 [Reference]	1 [Reference]	1 [Reference]
1% to ≤50% (weight gain)	0.86 (0.69-1.06)	0.89 (0.71-1.12)	0.85 (0.67-1.08)
−3% to ≤−1%	1.12 (0.88-1.41)	1.18 (0.92-1.51)	1.08 (0.83-1.41)
−5% to ≤−3%	1.36 (0.99-1.88)	1.31 (0.93-1.83)	1.28 (0.90-1.81)
−10% to ≤−5%	1.23 (0.82-1.85)	1.55 (1.02-2.36)	1.41 (0.91-2.17)
−50% to ≤−10%	2.27 (1.27-4.05)	0.67 (0.20-2.20)	1.08 (0.32-3.66)
Per 1% weight change	0.02 (0-0.15)	0.07 (0.01-0.97)	0.03 (0-0.51)
Odds adjusted for baseline weight and weight change interval length			
>−1% to ≤1%	1 [Reference]	1 [Reference]	1 [Reference]
1% to ≤50% (weight gain)	0.89 (0.71-1.10)	0.91 (0.72-1.15)	0.84 (0.66-1.07)
−3% to ≤−1%	1.14 (0.90-1.45)	1.23 (0.95-1.58)	1.08 (0.83-1.41)
−5% to ≤−3%	1.46 (1.06-2.02)	1.42 (1.01-1.99)	1.31 (0.92-1.86)
−10% to ≤−5%	1.48 (0.98-2.23)	1.72 (1.12-2.65)	1.47 (0.95-2.29)
−50% to ≤−10%	2.90 (1.58-5.31)	0.91 (0.30-2.78)	1.01 (0.28-3.63)
Per 1% weight change	1.00 (0.08-12.88)	0.04 (0-0.63)	1.00 (0.05-19.02)
**By data source**			
Numeric observed data			
Weight change present	1.26 (1.04-1.52)	1.21 (0.99-1.49)	1.19 (0.96-1.48)
≥1% vs <1% weight loss	1.26 (1.04-1.52)	1.21 (0.99-1.49)	1.19 (0.96-1.48)
≥3% vs <3% weight loss	1.30 (1.00-1.68)	1.19 (0.90-1.56)	1.25 (0.94-1.67)
≥5% vs <5% weight loss	1.18 (0.82-1.69)	1.06 (0.70-1.60)	1.16 (0.76-1.79)
≥10% vs <10% weight loss	1.71 (0.92-3.18)	0.49 (0.14-1.68)	0.72 (0.20-2.56)
*ICD*/NLP data			
Weight change present	8.53 (6.99-10.40)	7.98 (6.46-9.86)	7.61 (6.07-9.55)
≥1% vs <1% weight loss	8.53 (6.99-10.40)	7.98 (6.46-9.86)	7.61 (6.07-9.55)
≥3% vs <3% weight loss	8.52 (6.99-10.40)	8.00 (6.47-9.89)	7.61 (6.07-9.55)
≥5% vs <5% weight loss	8.54 (7.00-10.42)	8.04 (6.51-9.94)	7.63 (6.08-9.58)
≥10% vs <10% weight loss	8.53 (7.00-10.41)	8.08 (6.54-9.98)	7.68 (6.12-9.64)

Abbreviations: *ICD*, *International Classification of Diseases, Ninth Revision* and *International Statistical Classification of Diseases and Related Health Problems, Tenth Revision*; NLP, natural language processing; OR, odds ratio.

### Weight Changes Across Specific Time Intervals of Interest

Results from conditional logistic regression including interpolated weight data, controlling for baseline weight, indicated that most categories of weight change (loss or gain) within 6 months of the lung cancer diagnosis were significantly associated with being a case (Figure). Odds ratios of cancer diagnosis following weight gain ranged from 1.77 (95% CI, 1.33-2.35) for 1% to 3% gain up to 17.69 (95% CI, 8.68-36.07) for weight loss from 10% to 50%. However, over the quarters prior to the diagnosis or index date, weight gain showed no added risk. Weight loss continued to show significant associations with an increased risk of lung cancer for at least 6 months prior to diagnosis. At 6 months prior to lung cancer diagnosis, the odds of lung cancer for those with 5% to 10% weight loss are 3.37 (95% CI, 2.15-5.28); at 12 months, the odd ratios were 1.48 (95% CI, 0.80-2.74) for this group.

**Figure.**
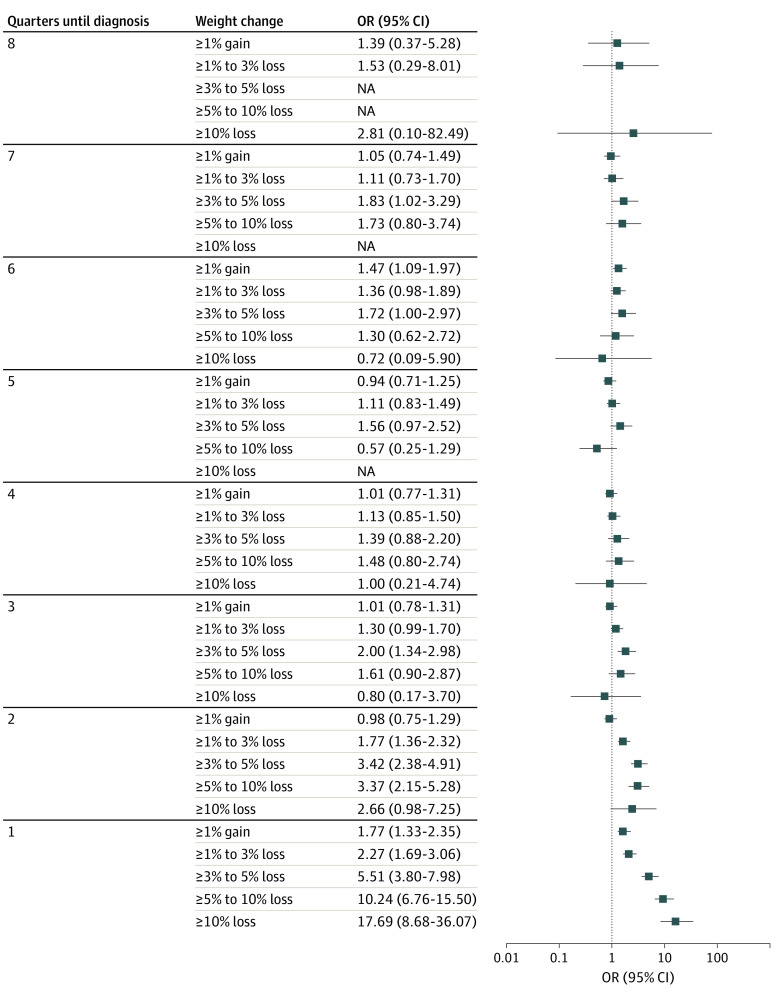
Odds of Lung Cancer Diagnosis by Quarter Prior to Diagnosis NA indicates not applicable; OR, odds ratio.

### Estimated Value of Measured vs *ICD* and NLP Weight Change Data

Table 3 demonstrates that as the observed weight loss from recorded data increased, the proportion of clinical notes that included an observation of weight loss in the free text or coded by *ICD* code increased from 49.2% for a 1% weight loss to 61.0% for a 5% or greater weight loss, holding at 59.3% for weight loss of 10% or greater. For controls, while there was an increase in documented weight loss in free-text notes corresponding with an increase in amount of weight loss, the majority of controls with substantial weight loss were not recorded in the notes.

**Table 3.** Agreement Between Recorded Clinical Observations From *ICD* or NLP Codes and Objective Weight Loss per Weight Measurement

Amount of weight loss	*ICD* code or NLP		*ICD* code only		NLP only	
	Cases	Controls	Cases	Controls	Cases	Controls
≥1%	273 (49.2)	420 (11.8)	92 (16.6)	189 (5.3)	261 (47.0)	280 (7.9)
≥3%	228 (55.5)	339 (15.2)	81 (19.7)	158 (7.1)	218 (53.0)	224 (10.0)
≥5%	153 (61.0)	228 (20.2)	59 (23.5)	114 (10.1)	145 (57.8)	148 (13.1)
≥10%	35 (59.3)	60 (24.6)	20 (33.9)	29 (11.9)	32 (54.2)	43 (17.6)

Abbreviations: *ICD*, *International Classification of Diseases, Ninth Revision* and *International Statistical Classification of Diseases and Related Health Problems, Tenth Revision*; NLP, natural language processing.

The odds of cancer for patients who had weight loss recorded as a code or free-text and measured weight loss were 1.3 times higher (OR, 1.26; 95% CI, 1.04-1.52) compared with patients who had coded or free-text weight loss but who did not have measured weight loss. Conversely, the odds of cancer for a patient who had weight loss noted as a code or free text and who had measured weight loss was 8.5 times higher (OR, 8.53; 95% CI, 6.99-10.40) compared with patients who had measured weight loss but who did not have weight loss noted in an *ICD* code or by free text. These ORs were smaller when the periods of time closest to diagnosis were excluded. In other words, clinicians’ explicit recording of weight loss (*ICD* codes or clinical notes) was a better estimate of subsequent lung cancer than a measured weight change.

## Discussion

### Main Findings

Using EHR data from a retrospective case-control study of individuals with primary lung cancer presenting in ambulatory care, our results indicated that in the 2-year period prior to diagnosis, individuals with lung cancer had greater odds of high levels of weight loss than matched controls. We also observed that clinicians’ recording of weight loss was more useful to estimate risk of an incident lung cancer diagnosis than measured weight change. Despite this, clinicians appeared to either miss or fail to record the presence of measured weight loss. We conclude that objectively measured changes in weight are apparently being missed, or at a minimum, are not recorded as noteworthy.

Cachexia is a well-recognized and very serious symptom of many different cancer types, and it is a particularly well-known syndrome at the time of diagnosis.^15^ Because of this, we did several analyses removing episodes immediately prior to diagnosis and confirmed that our findings were not merely confined to the period immediately prior to diagnosis when cachexia related to cancer may be occurring.

### Comparison With Previous Studies

While previous studies have noted associations between weight loss and cancer diagnosis in primary care settings, evidence from the US and for lung cancer specifically are sparse. A systematic review of 25 studies^16^ found that patients presenting to primary care with weight loss, either recorded by clinicians or by documented records of weight loss, were at higher risk of having several cancers, including lung cancer, than patients without recorded weight loss. However, none of these reviewed articles were based on EHR data in the US and all but 1 defined weight loss using a clinician entered code. In another subsequent large UK study of almost 64 000 patients whose primary care records were linked to cancer registry data, Nicholson et al^8^ reported an association between unexpected weight loss and the development of several cancers, including pancreas, cancer of unknown primary origin, gastroesophageal, lymphoma, hepatobiliary, bowel, kidney tract, and lung cancers. The only study we are aware of in US ambulatory care examining this association used data from Kaiser Permanente that found the risk of cancer diagnosis increased with percentage of weight loss and age (up to 85 years), as well as when the weight change interval decreased, with associations between weight loss and pancreatic, myeloma, gastroesophageal, colorectal, and breast (but not lung) cancers.^7^

### Implications for Clinicians, Researchers, and Policy Makers

While routine screening for lung cancer is recommended for higher risk individuals in the US, the vast majority of individuals are diagnosed after presenting with symptoms, or as incidental findings. Our research suggests that weight loss, identified from the routine weight measures or documented by clinicians as a symptom, is associated with lung cancer, even 3 or more months prior to diagnosis. Identifying individuals who may have lung cancer presenting to ambulatory care settings is challenging and is thought to contribute to the long periods of time many individuals with lung cancer experience prior to diagnosis, and potentially to the amount of malpractice claims. There is growing evidence that individuals later diagnosed with lung cancer often have a multi-month period of repeated visits to ambulatory care associated with evidence of distinct symptom signatures, as well as laboratory test abnormalities that differentiate them from patients without cancer in ambulatory clinics.^17,18,19^

However, translating associations noted from retrospective research studies into everyday clinical practice will require extensive implementation research. More data will be needed to populate risk estimation tools and inform clinical guidance from multiple data types, fields, and EHR systems in each international jurisdiction and then updated at repeated intervals. Moreover, decision support systems will need to be designed that complement clinical judgement for not only lung cancer, but potentially multiple other cancers. Research is needed to examine not only implementation but more importantly the impact on clinical decision-making (including the potential negative effects of overinvestigation and overdiagnosis), the identification of other serious illnesses, as well as evidence from prospective experimental studies of an impact on more timely diagnosis (or at an earlier stage of cancer), and ultimately improved clinical outcomes.

### Strengths and Limitations

We believe this is the first study to analyze the association of both measured weight loss and the symptom of weight loss recorded in the EHR with incident lung cancer diagnosis. This is a unique contribution of this work, as the comparison of the weight loss patterns picked up by the clinicians show an association with lung cancer diagnoses with high effect sizes, which is then contrasted with the recorded data showing a pattern of undiagnosed weight loss months prior to diagnosis. In addition, we show robust results with respect to acute effects, ie, eliminating results 1 and 3 months prior to diagnosis, when cancer-related cachexia may be escalating.

We note several limitations. First, cases were selected based on a diagnosis date defined as the date of the first lung cancer *ICD* code in the EHR, whereas controls were selected based on having a visit to the matched case clinic type (to account for difference in emergency vs other forms of ambulatory care) within 3 months of the case diagnosis date (to avoid potential seasonal differences in respiratory symptoms). Second, several cases and controls were eliminated from the analysis due to either missing weight data or changes in weight that were considered clinically implausible. While manual review of EHR could have been used to clarify or reduce these exclusions, we chose a more scalable approach to eliminating these. However, this implies either missingness of data, or errors in weight measures or recording that could limit generalizability of our findings. Third, availability of weight measures and recording of weight loss as a symptom among both cases and controls is reliant on the number of patient interactions with the health care system; those with more contacts (eg, due to comorbidities) may have had more data captured. There are 2 challenges to analyzing routinely collected data where patients have a variable number of visits and, therefore, a varying number of weight change episodes; different visit rates among cases than controls could have produced an uneven opportunity for weight change, and intraperson correlation among weight change episodes. We therefore randomly selected a single weight loss episode for each patient. Because we matched on smoking status, we could not include smoking directly in our models, nor could we formally compare models among smokers and nonsmokers. However, we did produce separate analyses to explore patterns of weight loss and case vs control strata within each group. The association we observed between weight loss and subsequent cancer diagnosis appeared to be roughly similar to sensitivity analyses with respect to smoking status, although there were some differences such as the coefficient for the percentage change in weight for smokers not being statistically significant when controlling for baseline weight and weight loss interval. This could be a function of sample size.

## Conclusions

In this case-control study of 625 patients with lung cancer and 4606 controls, we found that weight loss was associated with incident lung cancer and was present whether weight loss was recorded as a symptom by the clinician or based on changes in routinely measured weight, demonstrating a potential opportunity for early diagnosis. Added to the growing evidence of the unique symptom signature for lung cancer in the months prior to diagnosis, our findings support the need for research into the implementation of decision support to demonstrate benefits on patient outcomes.

## Notes

Supplement.
Data Sharing Statement
