# Supplementary material for: Association of Weight Loss in Ambulatory Care Settings With First Diagnosis of Lung Cancer in the US
Source: JAMA Netw Open. 2023 May 11;6(5):e2312042. doi: 10.1001/jamanetworkopen.2023.12042 (PMC10176120; doi:10.1001/jamanetworkopen.2023.12042)
Supplement: Supplement. — Data Sharing Statement [file jamanetwopen-e2312042-s001.pdf]

Data Sharing Statement

Kessler. Association of Weight Loss in Ambulatory Care Settings With First Diagnosis of Lung Cancer in the US. *JAMA Netw Open*. Published May 11, 2023.
doi:10.1001/jamanetworkopen.2023.12042

Data
Data available: Yes
Data types: Deidentified participant data, Data dictionary
How to access data: mjt@uw.edu
When available: With publication

Supporting Documents
Document types: None

Additional Information
Who can access the data: Researchers whose proposed use of the data has been approved.
Types of analyses: For any analysis approved by the authors.
Mechanisms of data availability: With investigators support and a signed data access agreement.
Any additional restrictions: None
